# Thermopriming reprograms metabolic homeostasis to confer heat tolerance

**DOI:** 10.1038/s41598-018-36484-z

**Published:** 2019-01-17

**Authors:** Natalia Serrano, Yu Ling, Ahmed Bahieldin, Magdy M. Mahfouz

**Affiliations:** 1 Laboratory for Genome Engineering, Division of Biological Sciences, 4700, King Abdullah University of Science and Technology, Thuwal, 23955-6900 Saudi Arabia; 2 Present Address: College of Agronomy, Guangdong Ocean University, Zhanjiang, 524088 China; 3 Department of Biological Sciences, Faculty of Science, King Abdulaziz University, P.O. Box 80141, Jeddah, 21589 Saudi Arabia

## Abstract

Heat stress threatens agriculture worldwide. Plants acquire heat stress tolerance through priming, which establishes stress memory during mild or severe transient heat stress. Such induced thermotolerance restructures metabolic networks and helps maintain metabolic homeostasis under heat stress. Here, we used an electrospray ionization mass spectrometry-based platform to explore the composition and dynamics of the metabolome of *Arabidopsis thaliana* under heat stress and identify metabolites involved in thermopriming. Primed plants performed better than non-primed plants under severe heat stress due to altered energy pathways and increased production of branched-chain amino acids, raffinose family oligosaccharides (RFOs), lipolysis products, and tocopherols. These metabolites serve as osmolytes, antioxidants and growth precursors to help plants recover from heat stress, while lipid metabolites help protect membranes against heat stress. The carbohydrate (e.g., sucrose and RFOs) and lipid superpathway metabolites showed the most significant increases. Under heat stress, there appears to be crosstalk between carbohydrate metabolism (i.e., the thermomemory metabolites stachyose, galactinol, and raffinose) and tyrosine metabolism towards the production of the thermomemory metabolite salidroside, a phenylethanoid glycoside. Crosstalk occurs between two glycerophospholipid pathways (the biosynthetic pathways of the thermomemory metabolite S-adenosyl-L-homocysteine and the terpenoid backbone) and the δ-tocopherol (chloroplast lipid) pathway, which favors the production of glycine betaine and other essential tocopherols, respectively, compounds which are essential for abiotic stress tolerance in plants. Therefore, metabolomic analysis can provide comprehensive insights into the metabolites involved in stress responses, which could facilitate plant breeding to maximize crop yields under adverse conditions.

## Introduction

Heat stress is one of the key abiotic factors which limit crop production and threaten food security. Plants respond to heat stress at the epigenomic, transcriptomic, epitranscriptomic, metabolomic, and proteomic levels^1,2^. Heat stress responses are conserved among eukaryotes, with both heat shock factors (HSFs) and heat shock proteins (HSPs) participating in these processes^3,4^. In general, HSFs regulate the transcription of HSP genes, while HSPs act as molecular chaperones to prevent the misfolding and denaturation of other proteins and help stabilize them under heat stress^5,6^.

Induced heat stress tolerance occurs during exposure to mild or severe and transient stress, resulting in the establishment of stress memory, which helps plants withstand subsequent severe stresses^7,8^. The initial stress leads to the priming for stress tolerance, and stress memory helps maintain tolerance to subsequent periods of stress^3,9^. In other words, induced thermotolerance occurs during subsequent exposure to high temperatures^3^. Recently, we developed a method for investigating the molecular basis of heat shock memory at the transcriptomic level and the role of priming in *Arabidopsis thaliana*^10^. This approach could be useful for increasing stress tolerance in crop plants during the growing season^11,12^, and would provide a better understanding of the molecular underpinnings of the thermopriming process.

Under abiotic stress, plants restructure their metabolic network to help maintain homeostasis via the production of stress-induced compounds. Hence, metabolomic analysis could provide comprehensive insights into the key metabolites involved in stress responses^13,14^. Data generated from such an approach will likely complement that derived from transcriptomic and proteomic analyses. Previous studies have shown that HSFs help to increase the levels of essential metabolites in plants under heat stress. HSFA2 and HSFA3 play roles in increasing the levels of the metabolite galactinol and its derivatives (e.g., raffinose family oligosaccharides [RFOs] such as raffinose and stachyose) in the cell in response to heat and oxidative stresses^12^. These metabolites help the cell to tolerate these types of stresses. One essential metabolite for oxidative stress tolerance, induced by heavy metals in Arabidopsis, is tocopherol (vitamin E). Previous screening of Arabidopsis plants for changes in lipid composition by thin-layer chromatography (TLC) indicated that tocopherol-deficient mutant line was tolerant to both wounding and heat stresses^15^. The authors indicated that other antioxidants can compensate for the loss of tocopherol. There are pathways that possibly crosstalk under heat stress. They include glycerophospholipid and terpenoid backbone biosynthesis pathways^16^. Thermomemory metabolites include S-adenosyl-L-homocysteine in the first pathway, and the plant chloroplast lipid δ-tocopherol in the second pathway. Pathway crosstalking can result in the production of important tocopherols that are crucial for the oxidative stress response in the membranes^17^. These related lipid metabolites are also involved in the protection of chloroplast lipids and chlorophyll as suggested in a study conducted on spinach and potato leaves, especially tocopherol^18,19^. Similarly, many other metabolites are accumulated or reduced in response to heat stress, but little is known about the metabolites behind the processes of thermopriming and thermomemory. It has long been suspected that thermopriming results from a complex cross talk between various processes controlling changes in membrane structure and function, water levels in the tissues, gene expression, lipid composition, proteins, and metabolite composition to help prepare plants to respond robustly and effectively to recurrent exposures to heat stress^16,20^.

In the present study, we explored the global composition and dynamics of the metabolome of Arabidopsis under heat stress in primed and non-primed plants. We have conducted metabolite profiling analysis to discover the thermomemory-related metabolites, resulting from heat stress-induced priming, using gas chromatography-mass spectrometry to detect the differential responses of metabolites to the thermopriming recently described by Ling *et al*.^3^.

## Results and Discussion

### Thermopriming regime for metabolomic analysis

**Figure 1:**
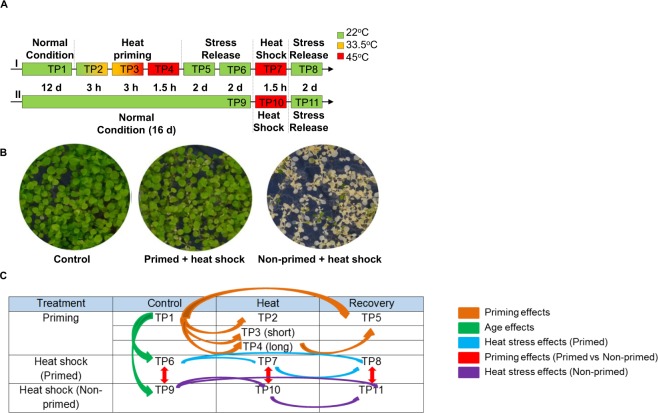
Heat stress priming and study design of global biochemical profiles comparison. (**A**) Outline of the heat stress-induced priming platform. (**B**) Phenotypes of Arabidopsis seedlings. Left, control plants without any treatment. Middle, plants experienced heat priming and then heat shock. Right, plants underwent heat shock only. (**C**) Study design of global biochemical profiles comparison.

We analyzed two sets of plants: heat primed and non-primed plants (Fig. 1A). For the primed set, we collected leaves from 12-d-old Arabidopsis seedlings before the initial exposure to heat stress (TP1). We then subjected these plants to heat stress-induced priming by exposing them to gradually increasing temperatures (from 22 °C to 45 °C) during a 6-h period, followed by 45 °C for an additional 1.5 h. Samples were collected 3 h (TP2) and 6 h (TP3) after initiation of the stress treatment, when the temperature reached 33.5 °C and 45 °C, respectively. Finally, we collected samples from plants after the final 1.5 h treatment at 45 °C (TP4). The latter heat stress regime was intended to induce the production of thermomemory metabolites in the primed set of plants. We transferred the remaining plants in this primed set to 22 °C for recovery (or stress release) and collected two samples: one after 2 days (TP5) and the other after 4 days (TP6). Concurrently, we collected leaf samples from 16-d-old non-primed plants (TP9) for use as a control to compare with those of primed plants (TP6), allowing us to differentiate between metabolites encoded by genes involved in thermomemory versus those encoded by genes that had not acquired thermomemory. We then exposed the plants to heat shock for 1.5 h and collected two samples: one from the primed set (TP7) and the other from the non-primed set (TP10). Finally, we maintained the heat-shocked plants at 22 °C for 2 days for recovery and collected two samples: one from primed (TP8) plants and the other from non-primed (TP11) plants. These two sets were used to investigate the differential responses of primed versus non-primed plants, which were evaluated after 6–10 d of recovery.

Overall, the primed plants were more vigorous compared with the non-primed plants, as the first set of plants had adapted to the second heat shock, after which thermomemory was established during the priming regime. The primed plants were able to survive normally after heat shock, while the non-primed plants started wilting five days after heat shock (Fig. 1B). The data were reproducible among three-replicate experiments, which were performed at different times. The study design for the comparison of global biochemical profiles is shown in Fig. 1C. Based on the findings of Ling *et al*.^3^, we speculated that thermomemory involves metabolites that are generated during the priming phase with a role in conferring memory-based heat stress tolerance. Therefore, we performed metabolic analysis of leaves collected at different time points (TP1–TP11).

### Heat priming and heat shock affect cellular metabolism

**Figure 2:**
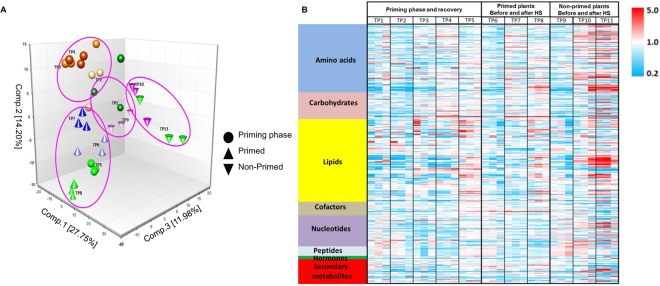
Principal component analysis (PCA) and global biochemical profiles at different time points and a heatmap based on metabolite abundances. (**A**) PCA showing group and separation of biochemical profiles between different time points. (**B**) Heatmap showing relative concentration of metabolites at different time points (TP), Columns represent the different samples, 3 biological replicates per sample. Rows the different compounds, in red more abundant and blue less abundant metabolites. “Heat priming” stands for exposure of plants to gradually increased temperature (primed plants). Non-primed plants are exposed to sudden heat shock treatment.

Principal component analysis (PCA) of thermoregulated metabolites grouped the control samples (TP1 and TP9). We used TP1 and TP9 as controls for the experiment and to detect age effect on leaves as shown in Fig. 2A and Table 1. These two samples grouped together suggesting the low variation in the metabolomic profile between them. TP8 and TP11 were separated from each other, as well as from the control samples. The results of PCA indicate that both primed and non-primed plants underwent active metabolic changes after heat shock. These changes were highly specific and were likely solely responsible for the different phenotypes of these plants. The changes in metabolic activity in plants suffering sudden heat shock in primed (TP7 versus TP6) and non-primed (TP10 versus TP9) plants were much smaller than those in the recovery phases of primed (TP8 versus TP6) and non-primed (TP11 versus TP9) plants, suggesting the long time required for metabolomic changes to occur (Table 1). Comparison between TP5 and TP8 indicated that the metabolic activities of these plants were highly similar (Fig. 2A). Across the 11 TPs, we detected 571 types of metabolites, including 150 amino acids, 59 carbohydrates, 182 lipids, 54 products of secondary metabolism, 68 nucleotides, 21 peptides, 7 types of hormone metabolites, and 30 cofactors, prosthetic groups, and electron carriers (Table 1). We constructed a heat map describing the relative abundance of the metabolites, revealing the disturbance of biochemical catabolism at different time points (Fig. 2B). Interestingly, plants in group TP11 (non-primed plants 2 d after HS) showed the most significant changes in metabolism (e.g., increased amino acid and lipid levels) compared to control plants at TP1 and TP9 (Fig. 2B). The results in Table 1 reveal a gradual increase in changes in metabolite levels (either up- or downregulated) in response to a gradual increase in heat stress (priming phase). During heat priming and recovery, 18, 36, and 59 metabolites showed altered levels at TP2, TP3, and TP4, respectively. At TP5 (2 d after stress release), the number of metabolites with altered levels continued to increase (102) but dropped to 34 at TP6 (4 d after stress release). We also detected increased numbers of metabolites with altered levels in primed and non-primed plants even after 2 d of stress release (TP5 versus TP4, TP8 versus TP7, and TP11 versus TP10). The number of metabolites with altered levels due to heat stress was higher in primed plants than in non-primed plants. These results point to the influence of the priming phase in stimulating cells to help them cope with subsequent exposure to heat shock due to thermomemory acquired during the earlier phase of treatment. The timing of heat stress initiation had little effect on this phenomenon (Fig. 1C).

**Table 1:** Number of the significantly disturbed metabolites in the different phases of the treatment.

Effects	Ratio	Up-regulated	Down-regulated	Total
Heat priming	TP2/TP1	2	16	18
	TP3/TP1	23	13	36
	TP4/TP1	33	26	59
	TP5/TP1	72	30	102
	TP5/TP4	139	77	216
Primed plants	TP7/TP6	79	60	139
	TP8/TP6	101	34	135
	TP8/TP7	106	64	170
Non-primed plants	TP10/TP9	79	32	111
	TP11/TP9	157	110	267
	TP11/TP10	12	29	41
Memory	TP6/TP9	29	11	40
	TP7/TP10	26	87	113
	TP8/TP11	104	121	225
Time	TP6/TP1	19	15	34
	TP9/TP1	12	20	32

Comparison between different time points during the stages of the treatment of primed and non-primed plants.

### Significant metabolic effects and responses during heat priming

**Figure 3:**
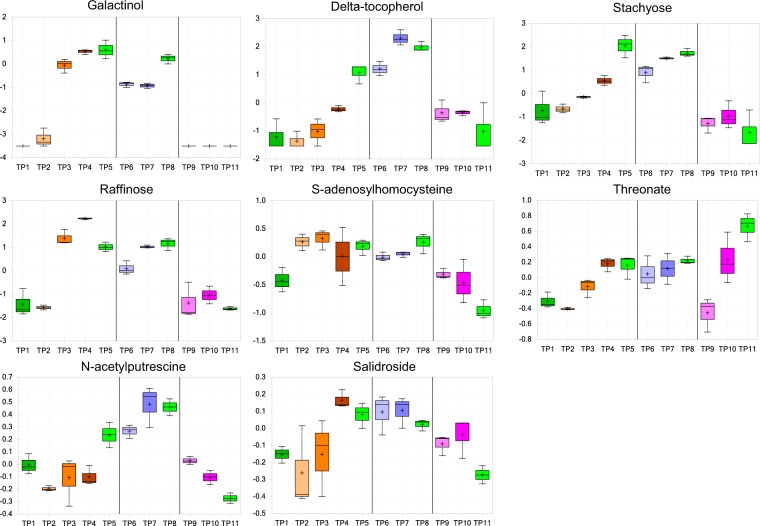
Memory metabolites accumulated during heat priming. Boxplots showing the levels of memory metabolites in all the different time points.

To investigate the major effects of heat priming on metabolism, we grouped metabolites whose levels increased ≥3 fold at TP4 compared with TP1 (Table 2); more than 12 compounds met this criterion. The metabolites with the highest increase in levels were in the carbohydrate superpathway, e.g., sucrose and RFOs, and those belonging to the lipids superpathway, e.g., 2-aminoheptanoate, glycerol 3-phosphate, and glycerophosphorylcholine (GPC). Membrane damage is an immediate effect of heat stress. However, previous studies indicated that moderate heat stress causes no apparent damage to membrane lipid structure^21^. Instead, stress alters cellular homeostasis to a suboptimal state that diminishes after stress release. Therefore, lipolysis that results in the production of glycerol backbones helps increase membrane stability under stress. RFOs and phospholipid backbones are strong osmolytes^22,23^. These findings suggest that the increases in sucrose, RFO, and lipid levels (which help protect membranes) represent a major response to heat priming. Such responses could help prepare primed plants for subsequent heat stress by allowing them to acquire thermomemory for several metabolites, such as galactinol, delta-tocopherol, stachyose, and raffinose (Fig. 3). Sucrose is a candidate signaling molecule for heat stress based on its early accumulation in response to high temperature^12^. Pathways that include sugar compounds may be important for the establishment and maintenance of acquired thermotolerance^10,24^.

**Table 2:** Major effects of heat priming regime in the main groups of metabolites.

Super Pathway	Sub Pathway	Biochemical Name	TP2/TP1	TP3/TP1	TP4/TP1	TP5/TP1	TP5/TP4
Amino acid	Aromatic amino acid metabolism (PEP derived)	3-(4 hydroxyphenyl)lactate	0.64	1.74	3.21^****^	1.01	0.32^**^
	Glutamate family (alpha-ketoglutarate derived)	Trans-urocanate	1.91	3.05	3.14	0.5^**^	0.16^*^
		Cis-urocanate	3.57	2.97^***^	3.84	0.87	0.23
	Glutathione metabolism	Ophthalmate	0.73	2.36	3.96	0.47	0.12^**^
Carbohydrate	TCA cycle	2-methylcitrate	0.93	2.25^****^	4.19^****^	5.17^****^	1.23
	Amino sugar and nucleotide sugar	UDP-N acetylglucosamine/galactosamine	1.65	2.96^***^	3.69^****^	1.19	0.32^**^
	Sucrose, glucose, fructose metabolism	1-kestose	1.18	4.33^****^	10.78^****^	2.14^****^	0.2^**^
		Galactinol	1.45	32.37^****^	56.6^****^	64.2^****^	1.13
		Raffinose	0.77	15.51^****^	34.51^****^	10.35^****^	0.3^**^
		Stachyose	0.9	1.48	3^***^	14.21^****^	4.73^****^
		Sucrose	0.29	10.95	45.69^****^	0.35	0.01^**^
Lipids	Fatty acid, Amino	2-aminoheptanoate	0.97	2.25^***^	3.07^****^	2.4^***^	0.78
	Phospholipid Metabolism	Glycerol 3-phosphate	0.85	2.82^***^	4.44^****^	3.04^***^	0.69^*^
		Glycerophosphoethanolamine	1.01	4.78^***^	4.57^***^	3.77	0.82
		Glycerophosphorylcholine (GPC)	1.19	10.6^****^	15.14^****^	14.42^****^	0.95
		Glycerophosphoserine	1.14	3.09^****^	3.15^****^	1.93^****^	0.61^*^
	Lyso-galactolipids	2-palmitoyl-galactosylglycerol (16:0)	1.59	3.38	4.6^***^	3.66^****^	0.8
Nucleotide	Purine metabolism	Adenylosuccinate	3.28	6.99^****^	3.74	0.34	0.09^**^

Chart showing the significant changes caused by raising the temperature during the priming phase, only compounds increased by ≥3-fold at TP4/TP1 are shown. Two pathways are drastically affected, carbohydrate and lipid, showing dramatic increases especially in the sub-pathways associated with sucrose and phospholipid metabolism. Data key: ****significant difference (p ≤ 0.05) between the groups shown, metabolite ratio of ≥1.00; ***narrowly missed statistical cutoff for significance 0.05 < p < 0.10, metabolite ratio of ≥1.00; **significant difference (p ≤ 0.05) between the groups shown, metabolite ratio of <1.00; *narrowly missed statistical cutoff for low significance 0.05 < p < 0.10, metabolite ratio of <1.00, No asterisks = no significance, showing metabolite ratio that missed statistical cutoff of p = 0.10 between the groups shown.

We investigated the effects of priming on heat shock stress in primed and non-primed plants before (TP6 versus TP9), during (TP7 versus TP10), and after (TP8 versus TP11) heat stress (Table 3). Several metabolites were more abundant in primed than in non-primed plants, before the heat shock treatment. These metabolites included RFOs, metabolites of branched-chain amino acid (BCAA) biosynthesis, and tocopherols. Four of these metabolites (galactinol, delta-tocopherol, stachyose, and raffinose) were defined as thermomemory metabolites, as they accumulated in response to heat priming and their levels continually remained higher in primed plants compared to non-primed plants; the two remaining metabolites are 2-isopropyl malate and dihydrokaempferol (Table 3). Several other metabolites also accumulated in primed plants during heat shock, including putrescine, 2-hydroxylaurate, glycerol 3-phosphate, and GPC. Twenty-one metabolites accumulated to high levels ( ≥ 5 fold) in primed plants and their levels increased more sharply during the recovery phase compared to those in non-primed plants (Table 3). These results demonstrate that primed plants maintain high levels of metabolites for energy pathways, BCAA biosynthesis, RFOs, lipolysis products, and tocopherols. The latter may serve as osmolytes, antioxidants, and precursors for new growth, which help plants survive and recover from the effects of lethal heat stress^25,26^. On the other hand, many metabolites strongly accumulated in non-primed plants during and after heat shock. These metabolites primarily include amino acid catabolites, RNA catabolites, lysolipids, and a few carbohydrates. The increased levels of many catabolism-associated compounds suggest that plants exhibit suboptimal growth under heat stress (Table 4).

**Table 3:** Accumulated metabolites in primed plants at recovery phase after heat shock, compared with non-primed plants.

Super pathway	Sub-pathway	Biochemical Name	TP6/TP9	TP7/TP10	TP8/TP11
Amino acid	Aspartate family (OA, A derived)	N-acetylmethionine	1.03	1.18	6.01^****^
	Branched chain amino acids (pyruvate derived)	2-isopropylmalate	6.33^***^	0.14^*^	9.68^****^
	Amines and polyamines	Putrescine	1.97	5.48^****^	13.16^****^
Carbohydrate	TCA cycle	Alpha-ketoglutarate	1.07	1.08	11.96^****^
		Fumarate	1.21	1.73	8.25^****^
	Amino sugar and nucleotide	N-acetylglucosaminylasparagine	1.03	2.13	6.7^****^
	Sucrose, glucose, fructose metabolism	Galactinol	14^****^	13.2^****^	42.17^****^
		Raffinose	3.64^***^	7.55^****^	16.35^****^
		Stachyose	8.8^****^	10.49^****^	23.53^****^
Lipids	Fatty acid, hydroxy	2-hydroxylaurate	1.65	5.04^****^	17.7^****^
	Fatty acid ester	Oleoylcholine	1.69	0.47	5.15^****^
	Phospholipid metabolism	Glycerol 3-phosphate	1.29	2.88^****^	6.22^****^
		Glycerophosphoethanolamine	1.35	3.93	30.55^****^
		Glycerophosphorylcholine (GPC)	3.03	10.9^****^	17.01^****^
		Glycerophosphoserine	0.95	1.55	5.46^****^
Cofactors	Tocopherol metabolism	Delta tocopherol	4.64^****^	14.6^****^	15.35^****^
Nucleotide	Pyrimidine metabolism	N-carbamoylaspartate	1.21	1.09	5.44^****^
Secondary metabolism	Flavonoids	Dihydrokaempferol	2.75^****^	3.27^***^	34.97^****^
		Naringenin	1.29	2.38	11.58^****^
	Phenylpropanoids	Sinapate	1.17	0.84	5.33^****^
		Lariciresinol	1.16	1.33	5.7^****^

Data key: ****significant difference p ≤ 0.05 between the groups shown, metabolite ratio of ≥1.00; ***narrowly missed statistical cutoff for significance 0.05 < p < 0.10, metabolite ratio of ≥1.00; *narrowly missed statistical cutoff for low significance 0.05 < p < 0.10, metabolite ratio of <1.00; No asterisk = no significance, showing metabolite ratio that missed statistical cutoff of p = 0.10 between the groups shown.

**Table 4:** Reduced metabolites in primed plants at recovery phase after heat shock, compared with non-primed plants.

Super pathway	Sub-pathway	Biochemical Name	TP6/TP9	TP7/TP10	TP8/TP11
Amino acid	Serine family (phosphoglycerate derived)	N-acetylserine	1.35^****^	0.48^*^	0.07^**^
	Aromatic amino acid metabolism (PEP derived)	Tyrosine	0.84	0.76	0.14^**^
		N-acetylphenylalanine	0.94	0.42^**^	0.05^**^
		N-formylphenylalanine	0.98	0.45^**^	0.11^**^
	Aspartate family (OAA derived)	N-acetylthreonine	0.71^*^	0.35^**^	0.11^**^
	Glutamate family (alpha-ketoglutarate derived)	4-hydroxybutyrate (GHB)	1.08	0.5^**^	0.08^**^
		N-acetylglutamine	1.53^****^	0.33	0.12^**^
	Branched chain amino acids (pyruvate derived)	N-acetylleucine	0.38	0.38^**^	0.07^**^
		N-acetylvaline	1.01	0.41	0.08^**^
Carbohydrate	Sucrose, glucose, fructose metabolism	Sucrose	3.07	1.82	0.04^*^
		Gluconate	1.23	0.37	0.15^**^
Lipids	Fatty acid, hydroxy	3-hydroxybutyrate (BHBA)	1.14	0.14^*^	0.03^**^
	Fatty acid, dicarboxylate	Undercanedioate (C11-DC)	1.14	0.31	0.14^**^
	Lyso-phospholipids	1-hexadecatrienoyl-GPA (16:3)	0.78	0.34^**^	0.06^**^
		1-linolenoyl-GPC (18:3)	0.8	0.3^**^	0.1^**^
		1-hexadecatrienoyl-GPG (16:3)	0.88	0.23^*^	0.09^**^
Nucleotide	Purine metabolism	2′-O-methyladenosine	0.66	0.54^**^	0.11^**^
		Cyclic(AMP-GMP)	0.67	0.09^**^	0.12^**^
	Pyrimidine metabolism	2′-O-methyluridine	0.84	0.5^*^	0.1^**^
	Dinucleotides	(3′-5′)-guanylyluridine	0.77	0.18^**^	0.15^**^
		(3′-5′)-uridylylguanosine	0.48	0.19^**^	0.1^**^

Data key: ****significant difference (p ≤ 0.05) between the groups shown, metabolite ratio of ≥1.00; **significant difference (p ≤ 0.05) between the groups shown, metabolite ratio <1.00; *narrowly missed statistical cutoff for significant 0.05 < p < 0.10 metabolite of <1.0; No asterisk = no significance, showing metabolite ratio that missed statistical cutoff of p = 0.10 between groups shown.

### Effects of heat priming at the pathway level

RFOs are sucrose molecules with α-1, 6-galactosyl extensions that are synthesized by a set of galactosyltransferases, which sequentially add galactose units from galactinol to sucrose (Fig. S1). Abiotic stress affects the expression of various genes responsible for RFO biosynthesis in plants^27^. The RFO pathway showed a high level of disturbance due to heat priming (Tables 2–4). Sucrose, mannitol/sorbitol, and 1-kestose levels increased to various extents under heat stress in both primed and non-primed plants. The levels of these compounds decreased after heat shock in primed plants, whereas they kept increasing after heat shock in non-primed plants (Fig. S1). The roles of these molecules in plants suffering from heat stress should be further evaluated.

**Figure 4:**
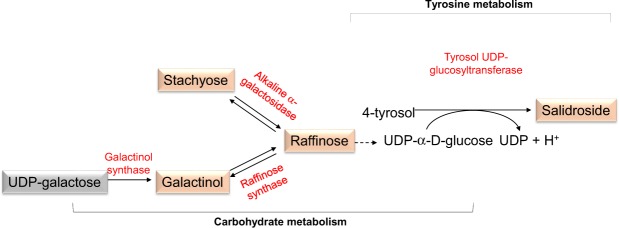
Regulation of some cross-talking memory metabolites of carbohydrate and tyrosine metabolisms pathways. A carton exploring potential metabolisms of crosstalk memory metabolites of carbohydrate and tyrosine metabolisms. Memory metabolites are shown in pink boxes.

Under heat stress, thermomemory metabolites involved in carbohydrate metabolism, including stachyose, galactinol, and raffinose metabolism, appeared to undergo crosstalk, as did tyrosine metabolism towards the production of the thermomemory metabolite salidroside, a phenylethanoid glycoside (Fig. 4). Salidroside is a primary bioactive marker in the medicinal plant *Rhodiola rosea*^28^. In the liver tissue of Sprague-Dawley rats, salidroside enhances the activity of antioxidant enzymes such as catalase, superoxide dismutase, and glutathione peroxide^29^. Salidroside also has protective effects against bone loss and exertional heat stroke-induced organ damage in model animals^30,31^. However, there is currently no substantial evidence for the possible role of this thermomemory metabolite in protecting plants against heat stress.

**Figure 5:**
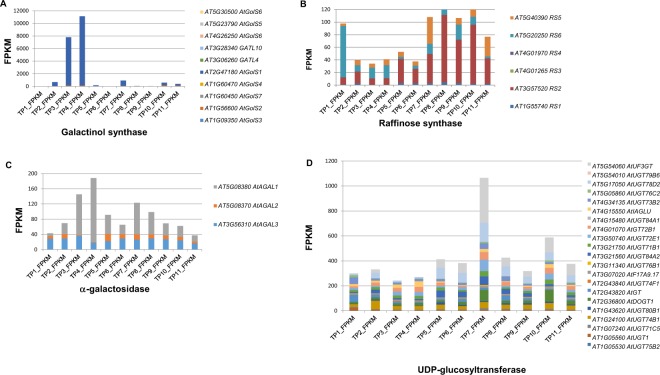
Transcriptional and post-transcriptional regulation RFO memory metabolite synthases. (**A**) FPKM values of galactinol synthase encoding genes at different time points. (**B**) FPKM values of raffinose synthase encoding genes at different time points. (**C**) FPKM values of α-galactosidase encoding genes at different time points. (**D**) FPKM values of UDP-glucosyltransferase encoding genes at different time points.

To investigate the response of these intersecting metabolic pathways, we examined data from our previous transcriptomic analysis^3^. We searched for genes encoding the metabolite synthases using gene IDs from TAIR (The Arabidopsis Information Resource; https://www.arabidopsis.org/). Interestingly, two galactinol synthase-encoding genes, *AtGolS1* and *AtGolS2*, were highly induced during the heat priming phase, as well as the heat shock phase (TP7 and TP10). However, these two genes exhibited significant intron retention (IR) at TP7, TP10, and TP11, which may have compromised their functional transcript levels at the lethal heat shock phase (Fig. 5A). The significant increases in *AtGolS1* and *AtGolS2* mRNA levels at TP3 and TP4 corresponded to the increasing levels of galactinol at the heat priming phase, which may have been partially sustained through TP5 and TP6. By contrast, the transcript levels of raffinose synthase genes did not significantly increase during the heat priming and heat shock phases (and those of *AtRS6* even decreased) in primed plants, suggesting that the high levels of raffinose in plants during and after heat priming were mainly due to the presence of a synthase other than raffinose synthase.

**Figure 6:**
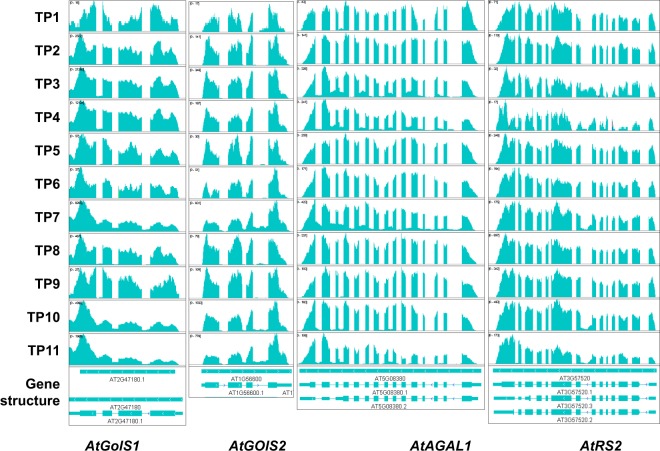
Post-transcriptional regulation of RFO memory metabolite synthases. Gene structure and intron retention profiles of *AtGolS1*, *AtGOIS2*, *AtAGAL1*, *AtRS2* showing alternative splicing patterns.

Interestingly, *AtRS2* and *AtRS6* were expressed at much higher levels at TP8 compared to TP11. Moreover, IR in *AtRS2* was detected at TP11, but not at TP8, which may have increased the difference between the amounts of functional mRNA for raffinose synthase after heat shock in primed versus non-primed plants. *AtAGAL1*, which undergoes IR under stress conditions, was the only galactosidase gene that was induced during heat priming and in primed plants (Fig. 6). This leads to the generation of higher amounts of galactosidase during heat priming and in primed plants compared to non-primed plants under the same conditions, which would correspond to the high levels of stachyose and raffinose in primed plants.

UDP-glucosyltransferases play a role in connecting carbohydrate metabolism and tyrosine metabolism. At the mRNA level, no significant difference was found during the priming phase for this group of genes compared to the control (TP1). Thus, some genes, including *AtUF3GT* and *AtUGT78D2*, were upregulated after heat priming and sharply upregulated in primed plants exposed to subsequent heat shock (TP7) compared to non-primed plants (TP10). The up-regulation of these few genes could have contributed to the significant quantities of UDP-glucosyltransferases detected in primed plants before, during, and after lethal heat shock, which corresponded to the high level of the thermomemory metabolite salidroside in primed plants. The high levels of induction of most of these genes were not sustained during the recovery phase (including TP5, TP6, TP8, and TP10), but their expression during the heat priming/heat shock phase could still have contributed to the generation of metabolites in the subsequent recovery phase; as mentioned above, the production of metabolites could be delayed due to the expression of these genes. Indeed, changes in the abundance of these thermomemory metabolites (Fig. 3) and several other metabolites in the RFO pathway (Fig. S1) correspond to the transcriptional and post-transcriptional regulation of related genes.

Phospholipid backbones, the products of lipolysis^21^, are essential compounds for membrane stability^32^ as these structures are composed mainly of lipids and proteins^33^. Heat stress affects the membranes by changing the fluidity and permeability^34^. The interaction between the hydroxyl groups of cholesterol with the phosphate of phospholipids is important to regulate membrane fluidity, packing, and formation of lipid microdomains^35^. The levels of phospholipids including glycerol 3-phosphate, glycerophosphorylcholine (GPC), and glycerophosphoethanolamine increased during heat priming, decreased at various rates during the recovery phase of priming, and remained stable or increased slightly in the subsequent heat stress and second recovery phase (Figs 3 and S2). As mentioned above, these results indicate that heat priming can protect the cellular membrane from being destroyed by heat due to lipolysis. This effect was sustained for several days, which would be beneficial for plants subjected to recurrent and transient heat stress.

**Figure 7:**
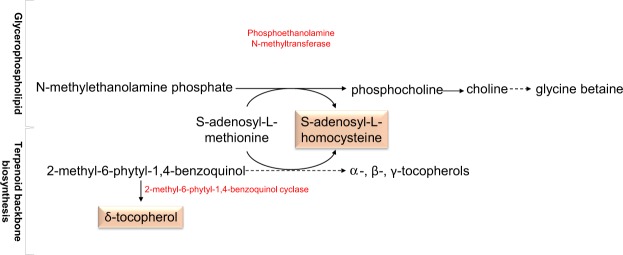
Crosstalk between glycerophospholipid and terpenoid backbone biosynthesis pathways. Metabolic reaction involving some memory metabolites of glycerophospholipid and terpenoid backbone biosynthesis pathways accumulated during heat priming towards the production of glycine betaine. Memory metabolites are shown in pink boxes.

Interestingly, the expression levels of *LCAT4* and *AT4G29070* (encoding proteins with phospholipase A activity) were high during the heat priming phase (Fig. S2B,C), pointing to the accumulation of phospholipid backbones and the increase in membrane stability, supporting previous studies that suggest a correlation between thermal stability of chloroplast membranes and the phospholipids^36^. The changes in the levels of these two proteins could initially have been due to transcriptional regulation during the heat priming phase. It appears that crosstalk occurs between two glycerophospholipid pathways (i.e., the biosynthetic pathways of the thermomemory metabolite S-adenosyl-L-homocysteine and the terpenoid backbone) and the pathway for the plant chloroplast lipid δ-tocopherol, which favored the production of glycine betaine and other essential tocopherols (or vitamin E), respectively (Fig. 7). The latter compounds are essential for conferring abiotic stress tolerance in plants^15,37^_._ Interestingly, glycine betaine is not a thermomemory metabolite per se; instead, it is a product of the thermomemory metabolite S-adenosyl-L-homocysteine.

The TCA cycle involves a group of chemical reactions used by all aerobic organisms to release stored energy through the oxidation of acetyl-CoA derived from carbohydrates, fats, and proteins into carbon dioxide and energy as adenosine triphosphate (ATP). The products of the TCA cycle include precursors of specific amino acids required for numerous biochemical reactions (Fig. S3). The TCA cycle is fundamental to many biochemical pathways. Pyruvate is the source of the TCA cycle, which leads to the production of acetyl-CoA^38^. The levels of pyruvate, together with two compounds in the TCA pathway namely fumarate and malate, increased slightly during heat priming and decreased to control levels during the recovery phase, followed by a slight increase in response to subsequent heat stress. Thus, in non-primed plants, the levels of three compounds of the TCA pathway namely pyruvate, malate, and fumarate, decreased in response to heat shock and further decreased during the recovery phase.

The levels of another compound of the TCA pathway, alpha-ketoglutarate, decreased in response to heat stress during priming and heat shock. In primed plants, alpha-ketoglutarate recovered to control or even slightly higher than control levels after the second heat shock, while in non-primed plants, its levels continually decreased after heat shock. In contrast to the compounds in the TCA pathway, the levels of lactate, which consumes carbon sources from the TCA pathway, highly increased in non-primed plants after heat shock and recovery but remained stable or slightly increased during heat priming and heat shock in primed plants. These results suggest that primed plants are highly efficient at producing energy when suffering from heat stress, whereas non-primed plants consume energy produced by the TCA cycle.

Most amino acids were present at similar levels in primed and non-primed plants, with a few exceptions: shikimate, 2-isopropylmalate, and putrescine (Fig. S4). Shikimate represents a primary carbon source acting as a precursor for the production of the three aromatic amino acids tryptophan, tyrosine, and phenylalanine in the pathway of amino acids biosynthesis. In addition, 2-isopropylmalate is a precursor for leucine production in the same pathway, while putrescine is a precursor for spermidine and spermine production in the arginine and proline metabolism pathway. The three aromatic amino acids, as well as spermidine, are active compounds in phenylpropanoid pathway, which is critical for growth through the production of flavonoids, coumarins, and lignans^39^. The latter compounds also act in defense mechanisms against pests such as insects, pathogenic fungi, and bacteria^40^. From our results, we can observe that shikimate was induced by heat shock in the primed plants but depleted in the non-primed plants. The levels of the leucine precursor 2-isopropylmalate decreased in response to heat treatment but increased during recovery only in primed plants. The leucine precursor 2-isopropylmalate showed an interesting behavior, as it was diminished by heat stress during the priming phase but was present at normal levels, similar to that of control sample, during the recovery phase. This behavior of 2-isopropylmalate was observed in the primed plants in TP7, the heat shock phase, but its level further increased even more than in TP6, the pre-stress phase. In contrast, the non-primed plants showed high levels of 2-isopropylmalate in TP10, but once the stress relieved, this compound was depleted.

Putrescine, spermidine, and spermine are natural polyamines that are essential for various cellular processes^41^, and their production is typically induced in anabolic cellular environments. These compounds are essential for many cell division processes during active growth, and they typically increase during growth stages. In our results, we observed their levels during the priming phase, which are maintained or increased in primed plants during recovery phase. However, they were depleted by heat shock or during recovery phase in non-primed plants (Table 3 and Fig. S4), suggesting that primed plants continue growing in part due to the increased levels of the polyamines.

Different changes in amounts of aromatic amino acids and polyamines, between primed- and non-primed plants, contribute to the performance of plants; e.g., primed plants survived after heat shock, while non-primed plants died after heat shock, indicating that accumulation of aromatic amino acids and polyamines may be stimulated by heat priming, but not after sudden severe heat shock.

Upon heat stress, the levels of antioxidant compounds, including glutathione (GSH), ascorbate, and tocopherols, were maintained in primed plants but not in non-primed plants (Fig. S5). The increased level of threonate, a catabolite of ascorbate, supports the idea that heat stress leads to the breakdown of ascorbate in non-primed plants, thus depleting the pools usually used to maintain glutathione reduction. By contrast, a parallel pathway producing antioxidant compounds were highly activated only in non-primed plants upon heat stress and its recovery phase. Except for indole-3-acetic acid, no phytohormones showed altered levels in primed plants in response to heat stress (Fig. S6). However, ethylene production was strongly induced by heat stress in non-primed plants, as the levels of cyano-alanine (a co-product of ethylene production) increased. Ethylene helps to protect plants from heat stress and/or repairs the accompanying damage from oxidative stress^42^.

### Relationship between metabolic reprogramming and heat tolerance

Heat stress in plants generally reduce growth, development, and reproduction, and at high temperatures can be lethal^43^. Thermopriming can mitigate the effects of heat stress in plants and make them tolerant to otherwise lethal temperatures^44^. This effect of priming is produced by a combination of many factors at different levels in the cell, and as we observed in this research, metabolic reprogramming contributes to the establishment of this thermomemory in plants. Our study shows that the priming effect involves the accumulation of metabolites, such as oligosaccharides of the sucrosyl-inositol pathway, glycerophosphocholine, TCA cycle energy intermediates, glutathione, ascorbate, tocopherols. These compounds have roles as osmolytes, energy intermediates and antioxidants mainly supporting the hypothesis that primed plants produce or accumulate higher levels of metabolites involved in anabolic metabolism, while non-primed plants suffered depletion of these compounds and instead accumulate many metabolites associated with catabolic metabolism. During the priming phase, the most affected metabolites were related to carbohydrate pathway especially the ones related to sucrose metabolism and the lipid pathway, with the phospholipid metabolism compounds the most affected. The metabolism of sucrose might contribute its carbon to several oligosaccharide pathways, including the formation of ketose. These oligosaccharides could possibly provide the primed plants with storage forms of carbon and play a role as osmolytes. The critical observation is that priming induces the Raffinose Family Oligosaccharide (RFO) pathways, and that the metabolites related to them maintain high levels in the first recovery phase and after heat shock in primed plants but not in non-primed plants. These data suggest that the RFO pathways play an essential role in the primed plants in response to heat stress.

The production of glycerol backbones from membrane phospholipids may also contribute and help primed plants to recover from heat stress. We found that phospholipid glycerol backbones precursors increased during the priming phase and remained high after recovery and even after heat shock in primed plants in comparison with non-primed plants indicating their important role in conferring thermotolerance.

## Conclusions

Heat stress, climate change, and the scarcity of water needed for irrigation pose significant threats to agriculture worldwide^45,46^_._ Heat stress retards plant growth, especially pollen development, hence markedly reducing grain yields^46–48^_._ Therefore, uncovering the molecular basis of plant responses and tolerance to heat stress will help breeders maximize crop yields in plants under adverse conditions^7,49–51^_._ One possible strategy for improving the plant’s ability to withstand heat stress is to stimulate the plant by moderate heat stress treatment to help it cope with subsequent exposure to normally lethal levels of heat stress^43^. This strategy, which is known as priming, allows plants to acquire thermotolerance, which is maintained over several days post-treatment^52–54^_._ Previous exposure to heat stress is thought to induce a response/adaptation to subsequent exposure to stress^8,55–58^. The term “priming” usually refers to a transient defense mechanism against pathogen attack that causes the plant to respond more efficiently to a future attack by the same pathogen^59^. The lag/memory phase that separates priming using moderate/gradual stress treatment from severe stress treatment is called the recovery phase. Recently, it has been demonstrated that heat stress priming is a result of splicing memory (leading to transcriptomic changes)^3^. Hence, plants utilize an efficient gene-splicing pattern that is maintained even after the stress is relieved. Temperature priming refers to several physiological and biochemical changes (mainly in membrane structure) that occur due to changes in primary and secondary metabolite composition^19^. In previous studies, it has been determined that Arabidopsis shoots begin to acquire thermotolerance at 40 °C; the level of tolerance increases with increasing exposure time (up to 4 h)^12^.

The ultimate goal of metabolomic analysis of plants under abiotic stress is to use the mechanistic knowledge gained in model organisms to unlock new approaches for breeding more heat-tolerant crop plants. Heat priming helps plants adapt to subsequent exposure to otherwise lethal levels of heat stress^44^. A recent study on the transcriptional and post-transcriptional regulation of gene expression during and after heat priming and heat shock stress showed that plants achieve “molecular thermomemory” at both the transcript splicing and alternative splicing levels^3^. The results of the current study suggest that heat priming re-balances metabolomic homeostasis. This process likely leads to an initial shift at the transcriptional level, which contributes to the establishment of a “thermo-memory” that provides primed plants with enhanced tolerance to repetitive heat stress.

## Methods

### Plant materials and growth conditions

*Arabidopsis thaliana* Col-0 wild-type seeds were sterilized using 10% bleach for 10 min, incubated at 4 °C for 2 days, and transferred to half-strength Murashige and Skoog medium agar plates with 1% sucrose in a growth chamber (Model CU36-L5, Percival Scientific, Perry, IA, USA) under long-day conditions: 16 h light (white light, ~75 µmol m^−2^ s^−1^) and 8 h dark, at 22 °C. The plants were divided into two groups for heat priming and no priming^3^.

### Heat priming and heat shock treatment

In order to investigate the metabolomic profile of primed and non-primed plants, we used a heat-priming design that we used in the previous analysis of the transcriptional and post-transcriptional regulation of heat stress priming and memory. This treatment consists of two groups of plants growing under the conditions previously described. One group is named primed and the second named non-primed plants. Initially, one sample is collected at 22 °C when the plants were 12 days old (TP1). Then, the primed plants were exposed to a gradual increase in temperature, priming phase, from 22 °C to 45 °C for 5 hours. And 2 samples were collected in this phase, time point 2 (33.5 °C) and time point 3 (45 °C), TP2 and TP3 respectively. Once the maximum temperature (45 °C) was reached, the plants were maintained for 1.5 hours at this temperature and a sample was taken at time point 4 (TP4) (45 °C). This first group of primed plants was placed again at 22 °C and kept with the non-primed plants. Two days after the priming phase, one sample is collected from the primed plants, time point 5 (TP5) (22 °C). Two days later, one sample at 22 °C was collected from each group, time point 6 (TP6) (primed-plants) and time point 9 (TP9) (non-primed plants) before exposing the plants of both groups to heat shock (45 °C for 1.5 hours). Immediately after the heat shock samples of each group were collected, time point 7 (TP7) and time point 10 (TP10), primed and non-primed plants respectively, at 45 °C. Both groups are placed back under normal growth conditions after heat shock, and two days later one sample of each group was collected, time point 8 (TP8) and time point 11 (TP11) at 22 °C, for primed and non-primed plants respectively. All samples were collected, ground in liquid nitrogen, frozen, and stored at −80 °C until the following analysis. Three biological replicates of each sample were collected.

### Sample preparation and metabolomic analysis

Sample preparation and metabolomic analysis were performed as described previously^60^. The samples were extracted with methanol under vigorous shaking for 2 min (Glen Mills GenoGrinder 2000) to precipitate the proteins and to dissociate small molecules bound to proteins or trapped in the precipitated protein matrix, followed by centrifugation to recover chemically diverse metabolites. The resulting extract was divided into five fractions: two for two separate reverse phase RP/UPLC-MS/MS analyses using positive ion mode electrospray ionization (ESI), one for RP/UPLC-MS/MS analysis using negative ion mode ESI, one for HILIC/UPLC-MS/MS analysis using negative ion mode ESI, and one that was reserved as a backup. The samples were briefly placed on a TurboVap (Zymark) to remove the organic solvent.

The samples were dried and reconstituted in solvents compatible with each of the four methods and analyzed by METABOLON, Inc. (North Carolina, USA). Each reconstitution solvent contained a series of standards at fixed concentrations to ensure proper injection and chromatographic consistency. For the UHPLC method, one aliquot was reconstituted in 50 µL of 0.1% formic acid in water, and the second aliquot in 50 µL of 6.5 mM ammonium bicarbonate in water, pH 8.0. For the HPLC method was used to reconstitute the aliquots 50 µL of 0.1% formic acid in 10% methanol. The reconstitution solvents contained internal instrument standards that were used to monitor the instrument and as retention index markers^60^. One aliquot was analyzed using acidic positive ion conditions, which were chromatographically optimized for more hydrophilic compounds. In this method, the extract was gradient-eluted from a C18 column (Waters UPLC BEH C18-2.1 × 100 mm, 1.7 µm) using water and methanol containing 0.05% perfluoropentanoic acid (PFPA) and 0.1% formic acid (FA). The second aliquot was also analyzed using acidic positive ion conditions but was chromatographically optimized for more hydrophobic compounds. In this method, the extract was gradient eluted from the aforementioned C18 column using methanol, acetonitrile, water, 0.05% PFPA, and 0.01% FA, which was operated at an overall higher organic content. The third aliquot was analyzed using basic negative ion-optimized conditions using a separate dedicated C18 column. The basic extracts were gradient-eluted from the column using methanol and water, but with 6.5 mM ammonium bicarbonate at pH 8. The fourth aliquot was analyzed via negative ionization following elution from a HILIC column (Waters UPLC BEH Amide 2.1 × 150 mm, 1.7 µm) using a gradient consisting of water and acetonitrile with 10 mM ammonium formate, pH 10.8. The MS analysis alternated between MS and data-dependent MSn scans using dynamic exclusion. The scan range varied slightly between methods but covered approximately 70–1000 m/z.

### Metabolite identification and data analysis

Compounds were identified via comparison to library entries of purified standards or recurrent unknown entities, as described by Evans *et al*.^60^. This library was based on authenticated standards containing the retention time/index (RI), the mass-to-charge ratio (m/z), and chromatographic data (including MS/MS spectral data) for all molecules present in the library. Additionally, biochemical identifications were based on three criteria: retention index within a narrow RI window of the proposed identification, an accurate mass match to the library +/− 10 ppm, and the MS/MS forward and reverse scores. The MS/MS scores were based on a comparison of the ions present in the experimental spectrum to ions present in the library entry spectrum. While there may have been similarities between these molecules based on one of these factors, the use of all three data points allowed us to distinguish and differentiate biochemicals. Additional mass spectra entries were created for structurally unnamed biochemicals, which were identified based on both chromatography and mass spectral analysis. Two types of statistical analysis were performed: (1) significance tests and (2) classification analysis. Standard statistical analyses were performed on log-transformed data using ArrayStudio. For analyses that were not standard for ArrayStudio, the program R (http://cran.r-project.org/) and JMP (SAS, http://www.jmp.com) a commercial software package was used. Significance tests used during this analysis included Welch’s two-sample *t*-test that was used to identify significant differences between experimental groups. Matched pairs *t-*test (one sample *t*-test), one-way ANOVA and two-way ANOVA were used for analysis of variances. P- (*p* ≤ 0.05) and Q-values were used to avoid false positive results^61^.

### Transcriptome data analysis

The transcriptome data was generated based on our earlier work^3^. Briefly, we ran the heat priming platform as we used in this study, and collected samples for RNA-Seq and following analyses. The annotated Arabidopsis gene models were downloaded from TAIR10 (https://www.arabidopsis.org/). TopHat (Version 2.0.10) was used for alignment and to predict splice junctions. Gene expression levels (FPKM value) were calculated using Cufflinks (Version 2.0.0).

## Electronic supplementary material

Supplementary information

## Data Availability

All data generated or analyzed in this study are included in this published article.
