# Supplementary material for: Thermopriming reprograms metabolic homeostasis to confer heat tolerance
Source: Sci Rep. 2019 Jan 17;9:181. doi: 10.1038/s41598-018-36484-z (PMC6336788; doi:10.1038/s41598-018-36484-z)
Supplement: Supplementary file 1 — Supplementary information [file 41598_2018_36484_MOESM1_ESM.pdf]

Supplementary Information

Article in *Scientific Reports*

Thermopriming reprograms metabolic homeostasis to confer heat tolerance

Natalia Serrano1, Yu Ling1,2, Ahmed Bahieldin3, and Magdy M. Mahfouz1*

[1]Laboratory for Genome Engineering, Division of Biological Sciences, 4700, King Abdullah University of Science and Technology, Thuwal 23955-6900, Saudi Arabia and [3]Department of Biological Sciences, Faculty of Science, King Abdulaziz University, P.O. Box 80141, Jeddah, 21589, Saudi Arabia

[*]**Corresponding author:** Magdy M. Mahfouz (magdy.mahfouz@kaust.edu.sa)

Current Address: [2]College of Agronomy, Guangdong Ocean University, Zhanjiang, 524088, China

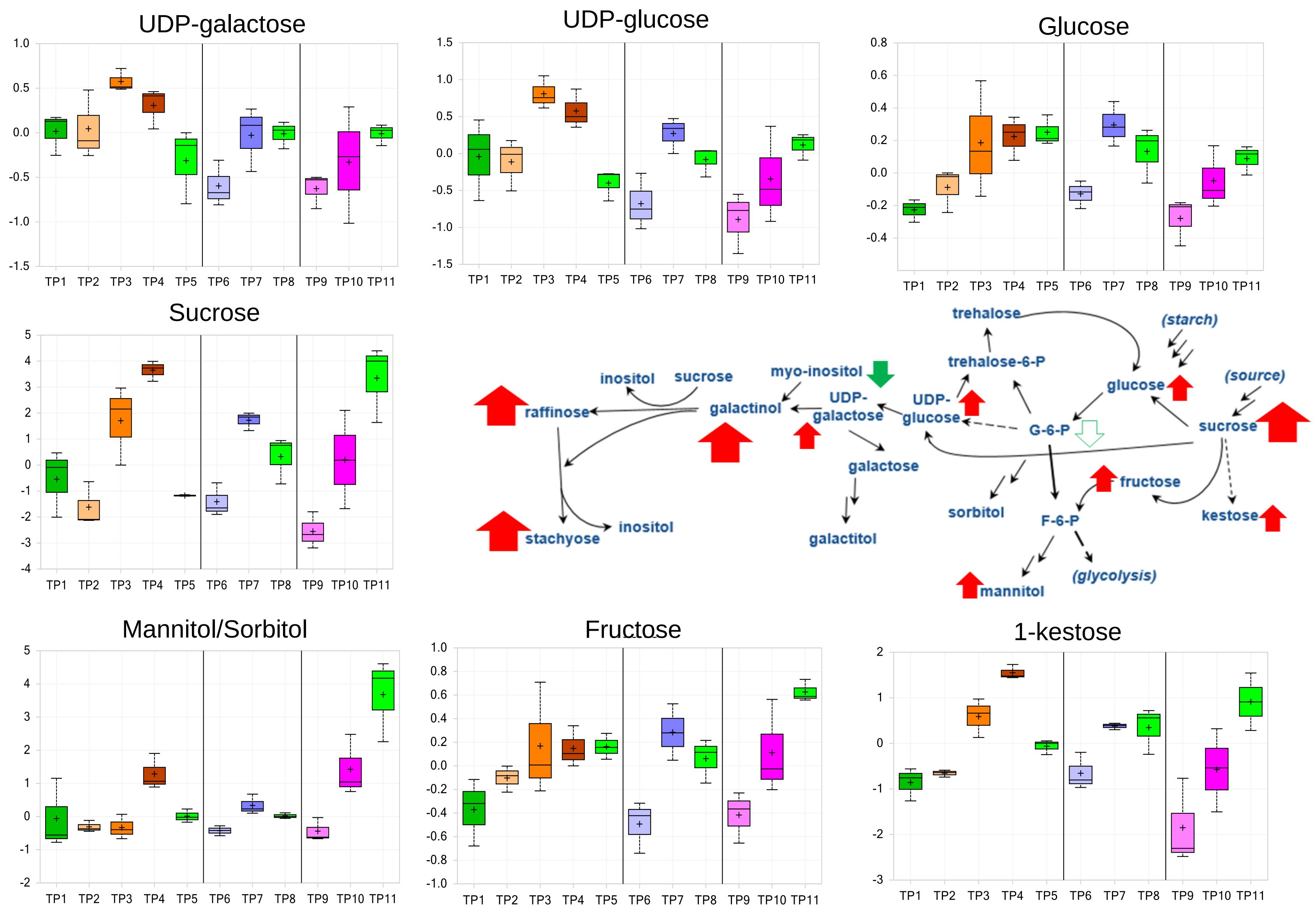

Figure S1. Effects of heat priming on RFO pathway. The RFO pathway is one of the most affected during the acclimation processes in Arabidopsis. Red arrows indicate the accumulated metabolites at the time points during the priming phase and green arrows the metabolites that decreases during this phase. Boxplots with the different patterns for major components of Oligosaccharide pathway during the different phases of priming and heat shock treatments.

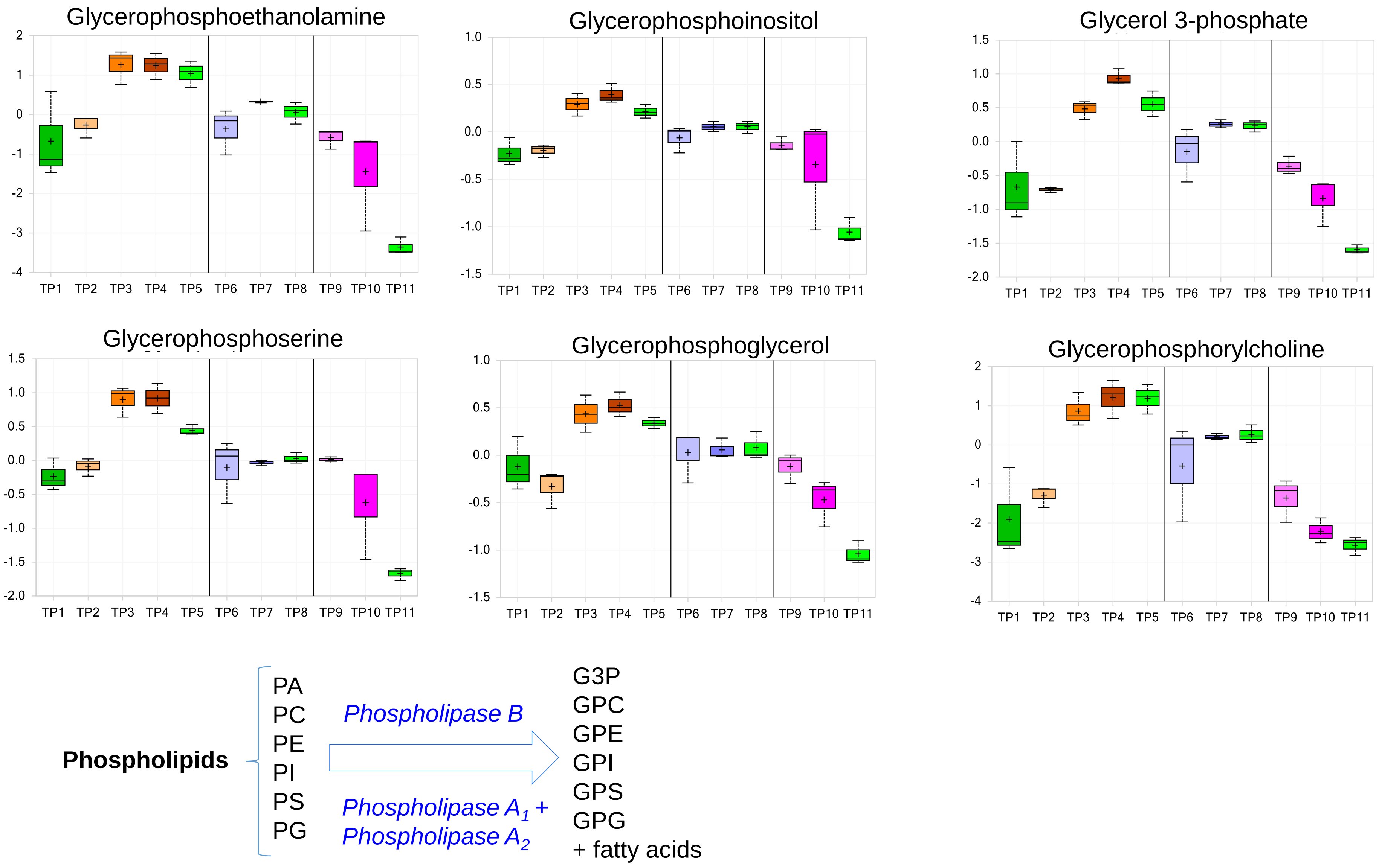

Figure S2. Effects of heat priming on lipolysis pathway. Boxplots with different metabolites product of the lipolysis elevated during the heat priming phase. Some of these products are either increased or maintained in the primed plants.

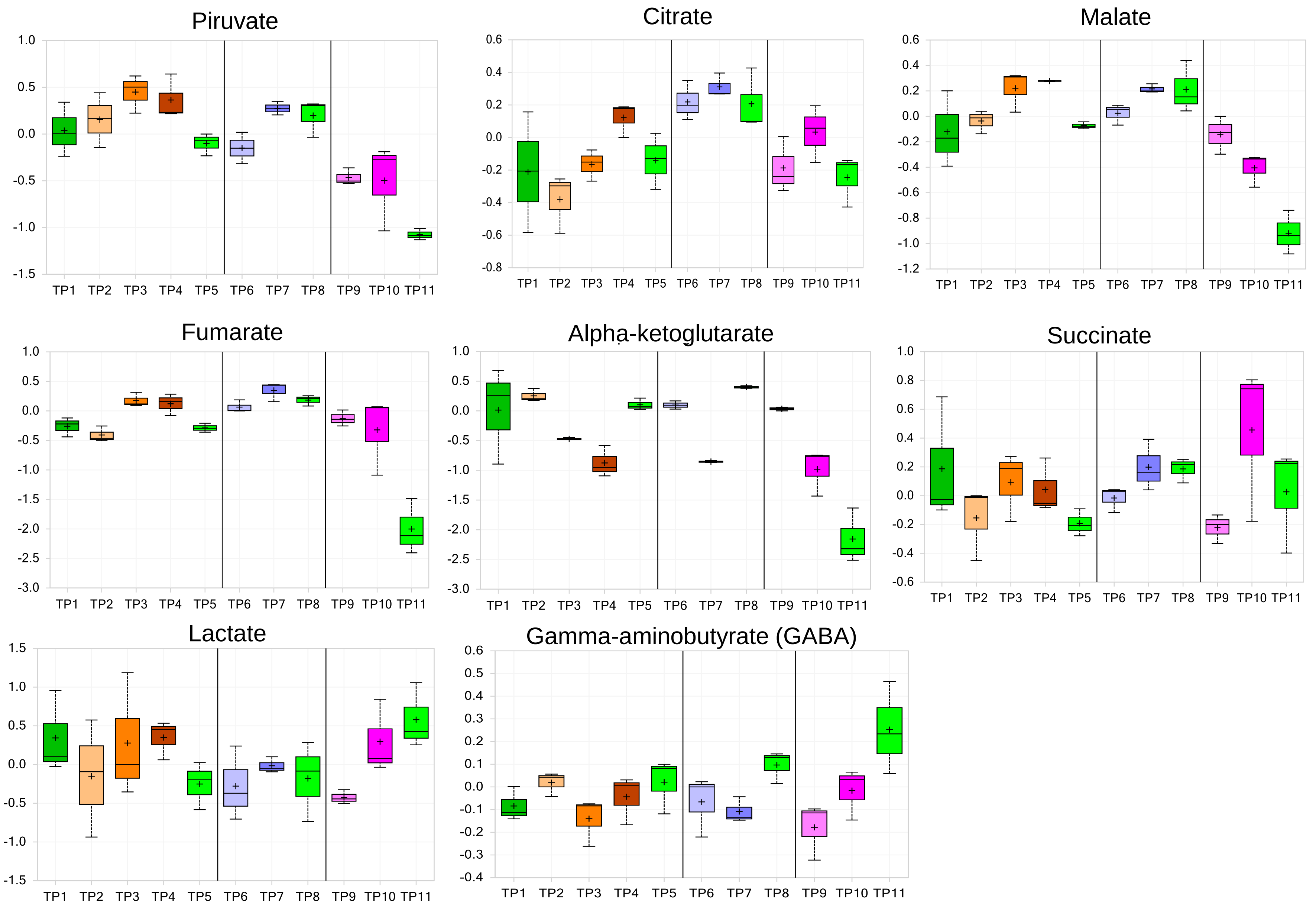

Figure S3. Effects of heat priming on energy pathway. Boxplots showing the different patterns for metabolites of the TCA cycle affected by heat priming.

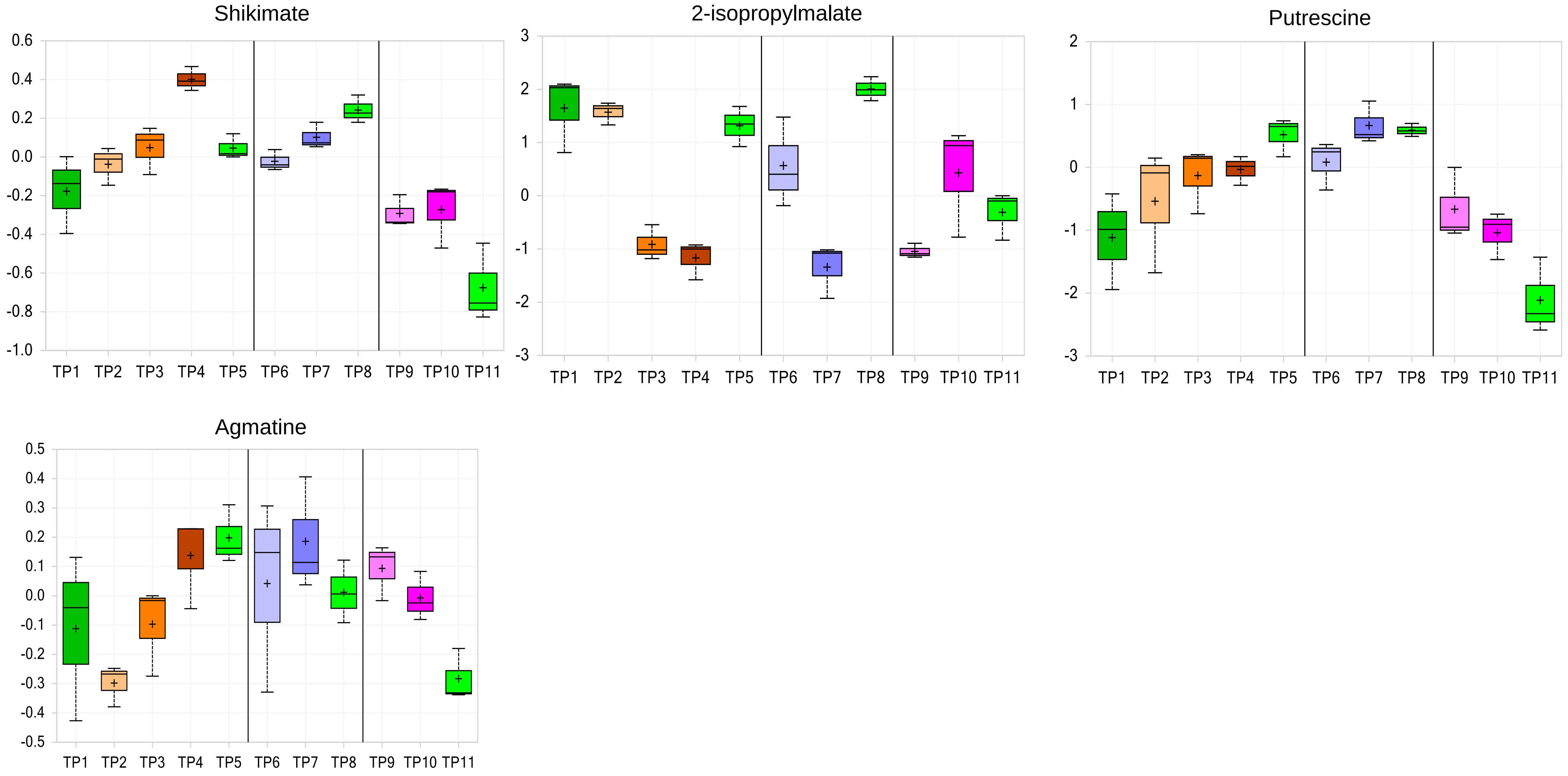

Figure S4. Effects of heat priming on amino acid pathway. Boxplots showing the different patterns for amino acids precursors in all time points.

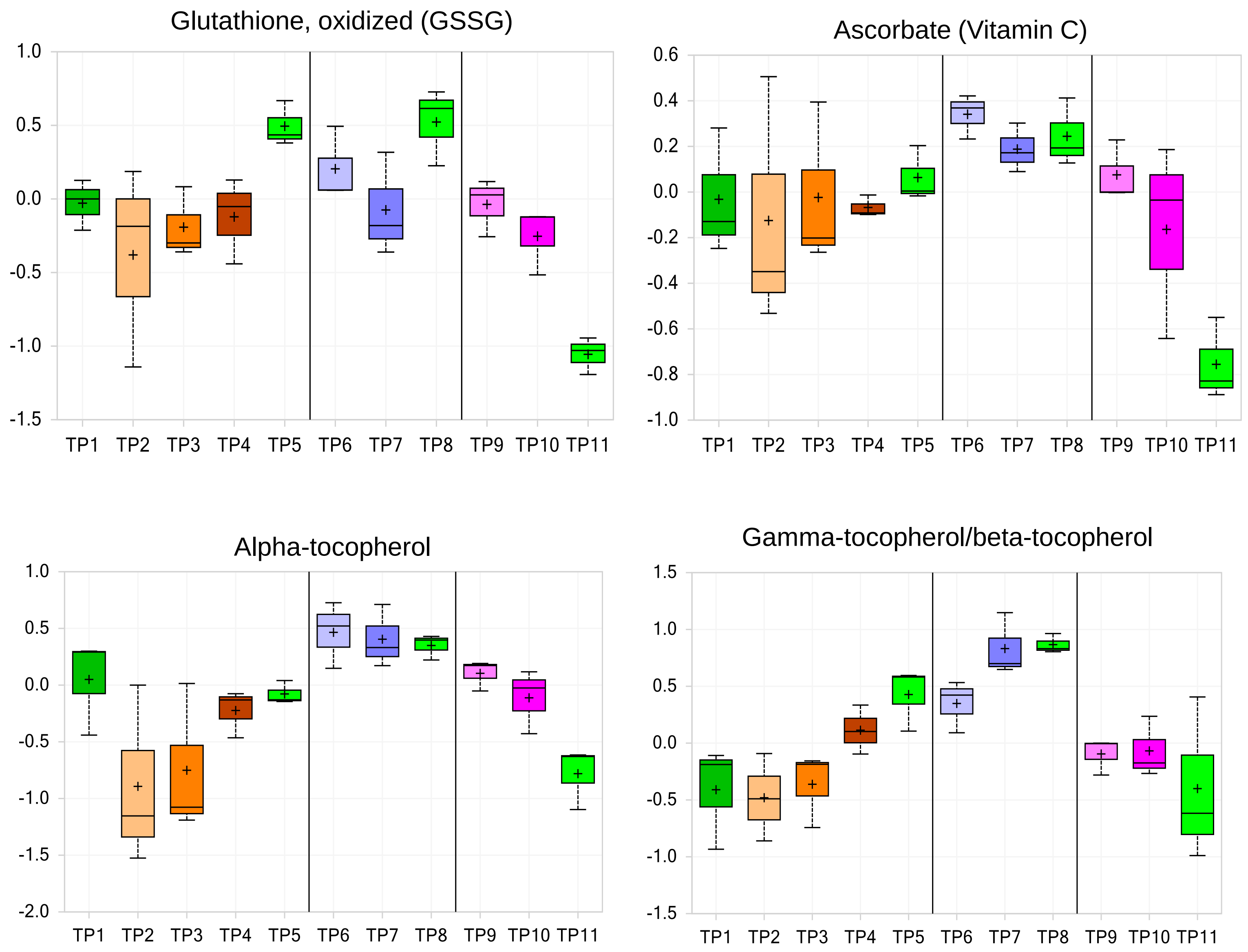

Figure S5. Effects of heat priming on antioxidant pathway. Boxplots of different metabolites of the antioxidant pathway affected in primed and non-primed plants.

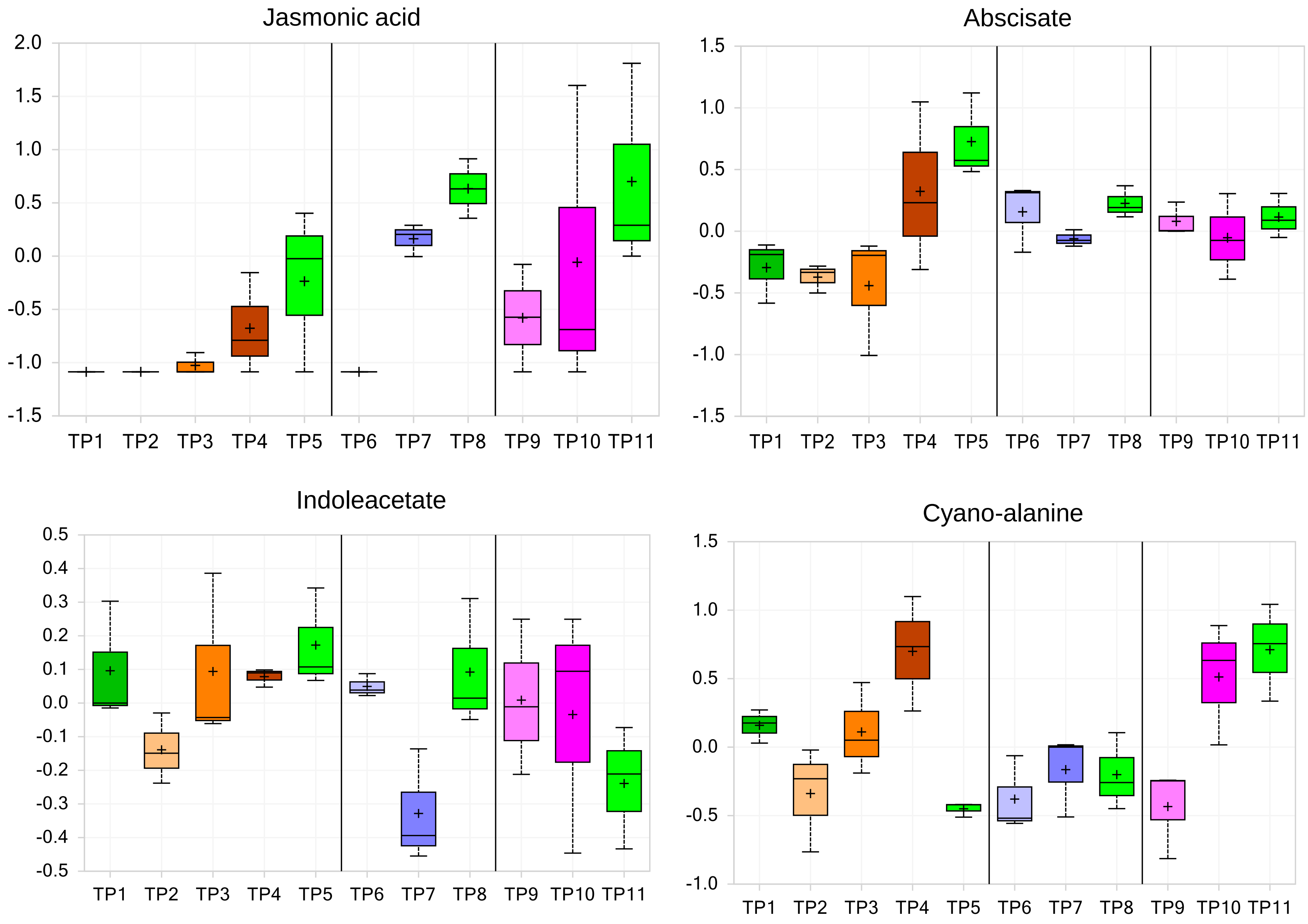

Figure S6. Effect of heat priming on phytohormone pathway. Boxplots showing the patterns of phytohormones with slightly increment during the priming phase.
